# Acetylome analysis reveals the involvement of lysine acetylation in diverse biological processes in *Phytophthora sojae*

**DOI:** 10.1038/srep29897

**Published:** 2016-07-14

**Authors:** Delong Li, Binna Lv, Lingling Tan, Qianqian Yang, Wenxing Liang

**Affiliations:** 1 The Key Laboratory of Integrated Crop Pest Management of Shandong Province, College of Agronomy and Plant Protection, Qingdao Agricultural University, Qingdao 266109, China; 2 College of Life Sciences, Qingdao Agricultural University, Qingdao 266109, China

## Abstract

Lysine acetylation is a dynamic and highly conserved post-translational modification that plays an important regulatory role in almost every aspects of cell metabolism in both eukaryotes and prokaryotes. *Phytophthora sojae* is one of the most important plant pathogens due to its huge economic impact. However, to date, little is known about the functions of lysine acetylation in this Phytopthora. Here, we conducted a lysine acetylome in *P. sojae*. Overall, 2197 lysine acetylation sites in 1150 proteins were identified. The modified proteins are involved in diverse biological processes and are localized to multiple cellular compartments. Importantly, 7 proteins involved in the pathogenicity or the secretion pathway of *P. sojae* were found to be acetylated. These data provide the first comprehensive view of the acetylome of *P. sojae* and serve as an important resource for functional analysis of lysine acetylation in plant pathogens.

Lysine acetylation is a reversible and highly conserved post-translational modification that plays an important role in almost every aspects of cell metabolism^1^. Lysine acetylation was first discovered in histone proteins^2^ and its role has been extensively investigated in regulating gene transcription^3^,^4^. Besides histones, many other proteins were also found to be acetylated^5^,^6^. Through reversible addition of an acetyl group to lysine residues, lysine acetylation regulates enzymatic activity, protein stability, protein localization and protein-protein and protein-nucleic acid interactions^7^,^8^. To comprehensively characterize acetylated proteins, lysine acetylomes have been determined for a number of eukaryotic and prokaryotic organisms^9^,^10^,^11^,^12^,^13^,^14^,^15^,^16^,^17^,^18^. These proteome-wide analyses of lysine acetylation reveal its broad roles in various cellular functions such as photosynthesis in plants, secondary and hormone metabolism, cell-cycle regulation, apoptosis and cell morphology^10^,^11^,^12^.

Increasing evidence suggests that lysine acetylation plays an important role during the invasion process of plant pathogens. For instance, effector HopZ1a, secreted by *Pseudomonas syringae*, has acetyltransferase activity. After being activated by the eukaryotic co-factor, phytic acid, HopZ1a can act on plant tubulin, and thus disrupts the plant secretory pathway and suppresses cell wall-mediated defense^19^,^20^. The *Ralstonia solanacearum* acetyltransferase effector, PopP2, directly acetylates a key lysine within an additional C-terminal WRKY transcription factor domain of RRS1-R that binds DNA, leading to altered immunity^21^,^22^,^23^.

Phytopthora forms a diverse group of fungus-like eukaryotic microorganisms that include some of the most important plant pathogens, *P. infestans*, *P. capsici*, *P. parasitica*, *P. ramorum* and *P. sojae*^24^. These pathogens are relatively closely related to photosynthetic algae such as brown algae and diatoms^25^,^26^,^27^. *P. sojae*, restricted primarily to soybean, causes enormous economic loss^28^. Despite its importance, actually little is known about the role of lysine acetylation in this plant pathogen. The presence of a large number of acetyltranferase and deacetylase orthologs in the genome of *P. sojae* suggests that lysine acetylation of proteins may play a critical role in this organism. To test this hypothesis, we performed the first large-scale analysis of lysine acetylated proteins in *P. sojae*. In total, 2197 lysine acetylation sites in 1150 protein groups were identified, which are involved in a variety of biological processes and multiple metabolism pathways. Importantly, several proteins involved in pathogenicity were found to be acetylated. This work provides the first extensive dataset on exploring the function of lysine acetylation in *P. sojae*.

## Results

### Identification and analysis of lysine-acetylated proteins in *P. sojae*

The large-scale dataset of lysine acetylation sites in *P. sojae* was determined using a proteomic method based on sensitive immune-affinity purification and high-resolution LC-MS/MS (Supplementary Fig. S1a). In total, 2197 lysine acetylation sites distributed in 1150 acetylated proteins were identified (Supplementary Table S1), which account for 6% (1150/19027) of the total proteins in *P. sojae*. To confirm the reliability of the MS data, the mass error of all the identified peptides were checked. The distribution of mass error is near zero and most of them are less than 5 PPM, which means the mass accuracy of the MS data fits the requirement (Supplementary Fig. S1b). Moreover, consistent with the property of tryptic peptides, the length of most peptides was distributed between 8 and 20 (Supplementary Fig. S1c). Thus, the data of modified peptide obtained from MS are of high accuracy.

### Functional annotation and cellular localization of acetylated proteins in *P. sojae*

**Figure 1:**
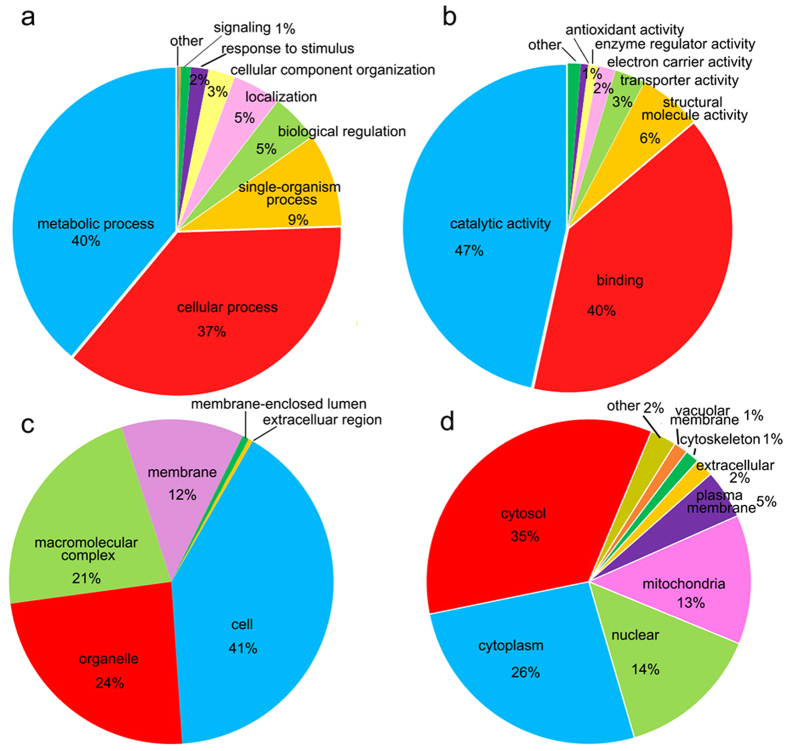
Functional classification of acetylated proteins in *P. sojae*. (**a**) Classification of the acetylated proteins based on biological process. (**b**) Classification of the acetylated proteins based on molecular function. (**c**) Classification of the acetylated proteins based on cellular component. (**d**) Subcellular localization of the acetylated proteins.

To gain further insights into the lysine acetylome of *P. sojae*, we investigated the Gene Ontology (GO) functional classification of all acetylated proteins based on their biological process, molecular function and cellular component (Fig. 1a–c, Supplementary Table S2). The biological process analysis indicates that most acetylated proteins were involved in metabolic processes (40%) and cellular processes (37%) (Fig. 1a). According to the molecular function classification, the largest group of acetylated proteins were composed of enzymes associated with catalytic activity, which accounts for 47% of all identified acetylated proteins. Another large group regarding molecular function consisted of proteins related to the binding of various targets (40%) (Fig. 1b). The results of cellular component analysis showed that the acetylated proteins identified were distributed in the cells (41%), organelles (24%), macromolecular complexes (21%) and membranes (12%) (Fig. 1c). Based on the subcellular localization predication, most of the acetylated proteins were distributed in the cytosol (35%), cytoplasm (26%), nucleus (14%) and mitochondria (13%) (Fig. 1d). Interestingly, 3 acetylated proteins were observed to be localized in the extracellular space. These results indicate that the acetylated proteins have diverse functions in *P. sojae*.

### Analysis of acetylated lysine sites

**Figure 2:**
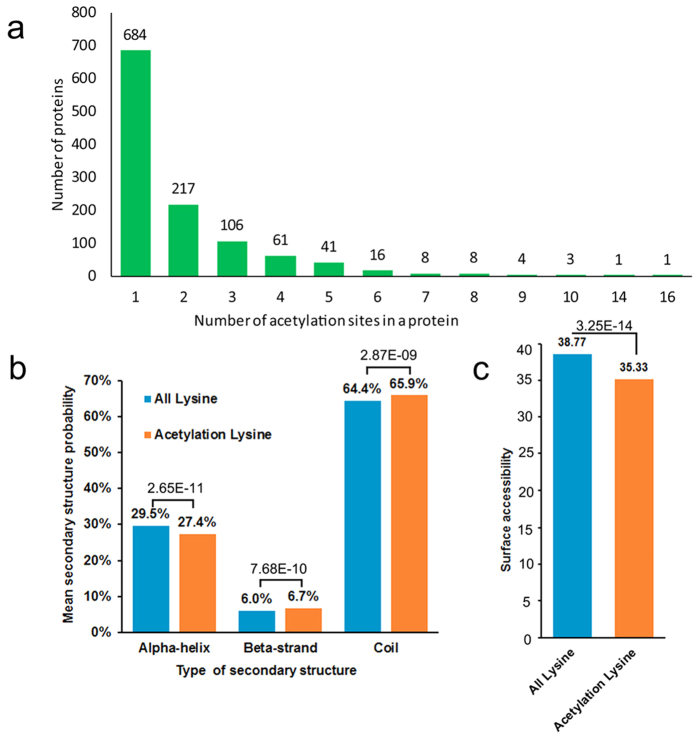
Properties of acetylated sites. (**a**) Distribution of acetylated sites in the acetylated proteins. (**b**) Probabilities of lysine acetylation in different protein secondary structures (alpha-helix, beta-strand and coil). (**c**) Predicted surface accessibility of acetylated sites.

To understand the property of lysine acetylation sites, the distribution of modification sites in each protein was examined. The results showed that the number of lysine acetylation sites in each protein was from 1 to 16 (Fig. 2a). From the results we found that approximately 60% of the proteins contained only one acetylation site, whereas 40% of the proteins were modified on multiple lysine residues. To explore the local secondary structures of lysine acetylation, a structural analysis of all the acetylated proteins was performed. As shown in Fig. 2b, 34.1% of the acetylation sites was located at regions with ordered secondary structures. Among them, 27.4% was located in α-helix and 6.7% in beta-strand (Fig. 2b). In addition, 65.9% of the acetylation sites was distributed in regions predicted to be disordered of proteins (Fig. 2b). These results suggest the tendency of acetylation on unstructured regions in *P. sojae* proteins. Surface accessibility of the acetylated lysine sites was also analyzed. The results in Fig. 2c showed that 38.8% of all lysine residues and 35.3% of the modified sites were located to the protein surface, respectively (*p* = 3.25 e–14). Therefore, lysine acetylation is likely to slightly affect the surface property of modified proteins in *P. sojae*.

**Figure 3:**
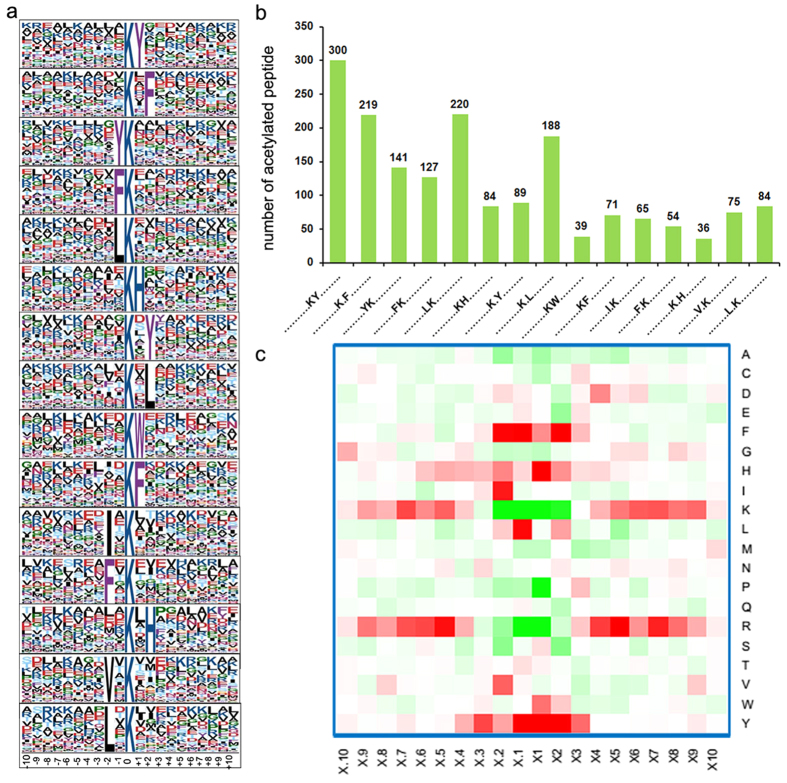
Properties of the acetylated peptides. (**a**) Acetylation motifs and conservation of acetylation sites. (**b**) Number of identified modification sites in each acetylated protein. (**c**) Heat map of the amino acid compositions of the acetylation sites. (red indicates enrichment and green means depletion)

To further identify a possible consensus sequence around acetylated lysine sites, the sequence motifs in all of the identified acetylated peptides were examined using Motif-X program. 2197 acetylated peptides, which include the amino acid sequence from the −10 to +10 positions around the acetylated lysine, were matched to fifteen conserved motifs (Fig. 3a, Supplementary Table S3). Particularly, motifs K^Ac^Y, K^Ac*^F, LK^Ac^ and K^Ac^*L occupied the highest proportion (K^Ac^ represents acetylated lysine and *represents a random amino acid residue). The acetylated peptides with these motifs were 300, 219, 220 and 188, which account for 26.1%, 19.0%, 19.1% and 16.3% of all the identified peptides, respectively (Fig. 3b). Consistent with our observations, most of the acetylation motifs identified in *P. sojae* were also found in other organisms^29^, confirming that lysine acetylation is a highly conserved modification among different species. According to the heat map of the amino acid compositions surrounding the acetylation sites, the frequency of tyrosine (Y) and phenylalanine (F) in positions −2 to +2 was highest, while the occurrence of K and arginine (R) was lowest (Fig. 3c). Based on these findings, we conclude that proteins with Y and F but without K and R around lysine residues will be preferred targets of lysine acetyltransferases in *P. sojae*.

### Functional enrichment analysis

**Figure 4:**
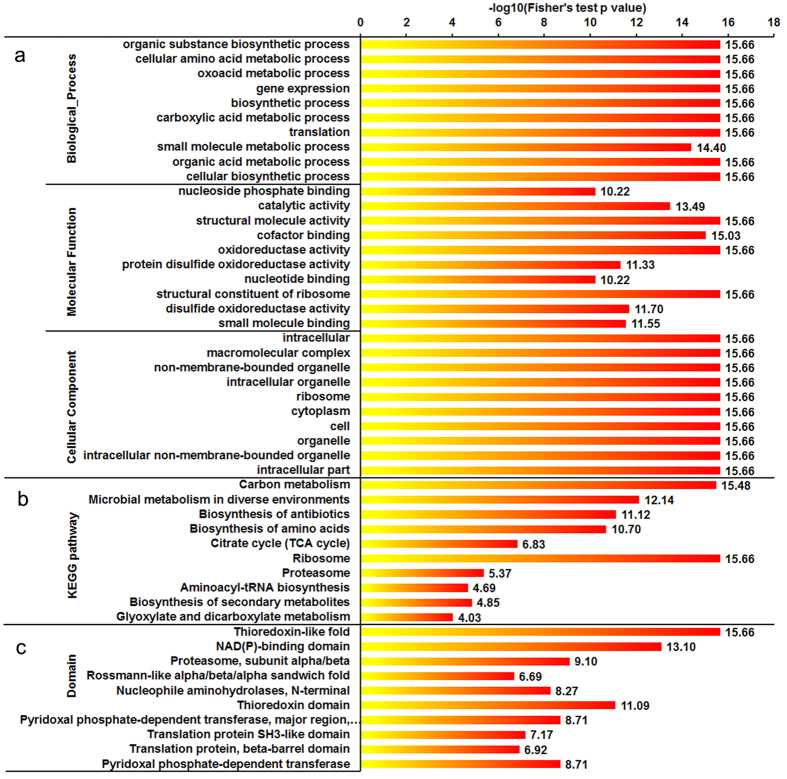
Enrichment analysis of the acetylated proteins in *P. sojae*. (**a**) GO-based enrichment analysis in terms of biological process, molecular function and cell component. (**b**) KEGG pathway-based enrichment analysis of the acetylated proteins. (**c**) Domain-based enrichment analysis of the acetylated proteins.

To understand the functionality of lysine acetylation proteins in Phytophthora, we performed GO (biological process, molecular function, and cellular component), KEGG pathway and protein domain enrichment analyses. In the biological process category, most of the acetylated proteins were shown to be involved in metabolic processes, gene expression and biosynthetic processes (Fig. 4a, Supplementary Table S4). In agreement with these observations, the enrichment analysis based on molecular function showed that many modified proteins were associated with binding and catalytic activity (Fig. 4a, Supplementary Table S5). Consistently, in the GO cellular component category, a large number of acetylated proteins were significantly enriched in the cytoplasm and intracellular space (Fig. 4a, Supplementary Table S6). In support of these observations, KEGG pathway enrichment analysis indicated that most of the acetylated proteins were related to metabolic pathway and biosynthesis (Fig. 4b, Supplementary Table S7). Remarkably, the enrichment of proteins involved in biosynthesis of secondary metabolites, biosynthesis of antibiotics and microbial metabolism in diverse environments suggests an important regulatory role of lysine acetylation in the ability of microbes adapt to stress conditions. Protein domain enrichment analysis revealed that proteins containing such domains as thioredoxin-like fold, NAD(P)-binding domain and thioredoxin domain, have higher tendency to be acetylated (Fig. 4c, Supplementary Table S8). Taken together, these data indicate that the acetylated proteins, widely distributed in the cell, are involved in a variety of processes in *P. sojae*.

### Protein-protein interaction network

**Figure 5:**
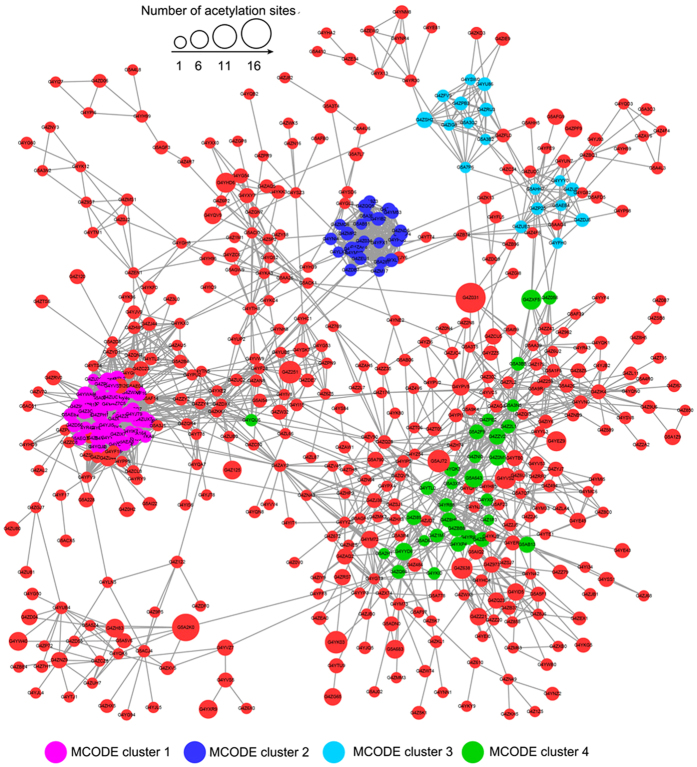
Interaction networks of acetylated proteins in *P. sojae*. Interaction networks of all acetylated proteins with the top four clusters of proteins associated with ribosome (pink), proteasome (dark blue), oxidative phosphorylation (blue) and aminoacyl-tRNA biosyntheisis (green).

Many molecular processes within a cell involve a large number of protein components, and the interaction among these proteins is important for the cells^30^,^31^,^32^. To investigate cellular processes regulated by acetylation in *P. sojae*, we established the protein-protein interaction network (Fig. 5). A total of 564 acetylated proteins were mapped to the protein interaction database (Supplementary Table S9, Supplementary Table S10), which presents a global view of how acetylated proteins perform various types of functions in *P. sojae*. In total, 26 highly interconnected clusters of acetylated proteins were retrieved (Supplementary Table S9). The top four clusters identified include proteins associated with ribosome, proteasome, oxidative phosphorylation and aminoacyl-tRNA biosynthesis (Supplementary Fig. S2). The complicated interaction networks of acetylated proteins indicate that the physiological interactions among these protein complexes are likely to contribute to their cooperation and coordination in this important plant pathogen.

### Analysis of acetylated proteins involved in pathogenicity of *P. sojae*

As an important plant pathogenic oomycete, *P. sojae* employs a variety of strategies to infect plants. From the results, we found some proteins involved in the pathogenicity of *P. sojae* (Table 1) were identified to be acetylated, including heat shock transcription factor 1 (HSF1), protein disulfide isomerase (PDI), effectors (cellulose-binding elicitor lectin CBEL and avirulence gene Avr1d), secretion related proteins (SNARE YKT6 and small GTP) and signaling pathway (cAMP, MAPK). Among these proteins, PsHSF1 is critical for pathogenicity in *P. sojae* by detoxifying the plant oxidative burst. Reactive oxygen species (ROS) produced in plant defense can be detoxified by extracellular peroxidases and laccases which might be regulated by PsHSF1^33^. It is well known that secreted proteins are factors that can be recognized by the innate immune system of the host, leading to disease resistance. PpPDI1, a typical PDI protein from *P. parasitica*, is able to induce strong cell death in *Nicotiana benthamiana* leaves. Deletion analysis showed that the first CGHC motif in the active domain of PpPDI1 is essential for this function. Moreover, as a secreted protein, PpPDI1 is conserved in eukaryotes^34^. CBEL, a cell wall-associated glycoprotein elicitor of *P. parasitica*, could induce necrosis, ethylene biosynthesis, HRGP accumulation, peroxidase activity and expression of the defense-related genes *ACO1*, *ASA1*, *Wak1* and *PR-1* in Arabidopsis^35^. Avr1d, the product of the avirulence gene *avr1d* of *P. sojae*, could specifically trigger cell death in Rps1d soybean plants^36^.

Effectors secreted by plant pathogens have been implicated in disease symptoms in their hosts. We found that several key secretion proteins were acetylated. For instance, PsYKT6, a conserved SNARE protein, plays key roles in proteins secretion, asexual development, sexual reproduction and pathogenesis on host soybean cultivars^37^. The dynamin-like GTPase, vacuolar protein sorting 1 (Vps1p), is involved in the budding of vesicles transporting vacuolar cargo from the Golgi apparatus^38^. Inactivation of PsVPS1 not only affects cyst germination and the polarized growth of germinated cysts, but also impairs invasion ability of susceptible soybean plants regardless of wounding^39^.

Collectively, these data, together with those presented above, strongly suggest that lysine acetylation plays an important role in *P. sojae* pathogenicity.

## Discussion

Lysine acetylation is a dynamic and widespread post-translational modification in both prokaryotes and eukaryotes with diversified functions. Although known for many years, its role in pathogenicity of plant oomycete is elusive. In this study, we determined the acetylome of *P. sojae* with the identification of 2197 lysine acetylation sites in 1150 proteins, which account for 6% of the total proteins in *P. sojae* proteome. The modified proteins exhibit multiple cellular localizations and participate in a variety of biological processes. Importantly, several proteins related to pathogenicity were found to be acetylated. These findings widen the roles of reversible acetylation in *P. sojae* and open up new possibilities for investigations in the field.

In this research, we found some proteins related to pathogenicity of *P. sojae* were modified by acetyl groups, implying an important regulatory role of lysine acetylation in this process. In agreement with our observations, in *Fusarium graminearum*, the causal agent of Fusarium head blight in wheat and barley, it was shown that histone deacetylase, FTL1, plays a critical role in the penetration and colonization of wheat tissues^40^. Inactivation of two acetoacetyl-CoA acetyltransferases in *Magnaporthe oryzae*, MoAcat1 and MoAcat2, leads to defect in virulence^41^. Interestingly, some acetyltransferases and deacetylases were also observed among the acetylated proteins in this study and in other proteomic research^42^,^43^. All these findings strongly suggest that lysine acetylation is important for virulence of plant oomycete.

In conclusion, these results represent the first extensive data on lysine acetylation in *P. sojae*. Although these findings suggest an important regulatory role of lysine acetylation in the pathogenicity of *P. sojae*, additional experiments will be needed to conclusively prove this interesting phenomenon.

## Methods

### Protein extraction and trypsin digestion

Mycelium, the vegetative part and the pathogenic form of *P. sojae*, was collected and ground in liquid nitrogen. The sample was transferred to 5 mL lysis buffer (8 M urea, 1% Triton-100, 65 mM dithiothreitol (DTT), 1% Protease Inhibitor Cocktail, 3 μM trichostatin A and 50 mM nicotinamide) and sonicated three times on ice^44^,^45^. Note that lysine deacetylase inhibitors, trichostatin A and nicotinamide, were added to prevent deacetylation of acetylated proteins. Cell debris was removed by centrifugation at 12,000 g at 4 °C for 10 min. Finally, the protein was precipitated with 15% cold TCA for 2 h at 4 °C. After centrifugation at 4 °C for 10 min, the supernatant was discarded, and the precipitate was washed with cold acetone for three times. The protein was redissolved in buffer (8 M urea, 100 mM triethylammonium bicarbonate, pH 8.0), and protein concentration was determined with 2-D Quant kit (GE Healthcare) according to the manufacturer’s instructions.

The protein solution was reduced with 10 mM DTT for 1 h at 37 °C and alkylated with 20 mM iodoacetamide for 45 min at room temperature in darkness. The protein sample was diluted with 100 mM (NH_4_)_2_CO_3_ to urea concentration less than 2 M. Finally, trypsin (Promega) was added at 1:50 trypsin-to-protein mass ratio for the first digestion overnight and 1:100 trypsin-to-protein mass ratio for a second 4 h-digestion.

### HPLC fractionation

The sample was fractionated by HPLC using Agilent 300 Extend C18 column (5 μm particles, 4.6 mm ID, 250 mm length). Briefly, peptides were first separated with a gradient of 2% to 60% acetonitrile in 10 mM ammonium bicarbonate (pH 10) over 80 min into 80 fractions, Then, the peptides were combined into 6 fractions and dried by vacuum centrifuging.

### Affinity enrichment

Tryptic peptides were dissolved in NETN buffer (100 mM NaCl, 1 mM EDTA, 50 mM Tris-HCl, 0.5% NP-40, pH 8.0) and incubated with pre-washed anti acetyllysine antibody agarose beads (PTM Biolabs) at 4 °C overnight with gentle shaking. The beads were washed four times with NETN buffer and twice with ddH_2_O. The bound peptides were eluted from the beads with 0.1% trifluoroacetic acid and vacuum-dried. Prior to LC-MS/MS analysis, the obtained peptides were rinsed with C18 ZipTips (Millipore) according to the manufacturer’s instructions.

### LC-MS/MS analysis

Peptides were dissolved in 0.1% formic acid (FA) and separated by a reversed-phase analytical column (Acclaim PepMap RSLC, Thermo Scientific). The gradient was comprised of an increase from 7% to 18% solvent B (0.1% FA in 98% acetonitrile) for 16 min, 18% to 22% for 8 min, 22% to 35% for 8 min and climbing to 80% in 5 min then holding at 80% for the last 3 min, all at a constant flow rate of 300 nl/min on an EASY-nLC 1000 UPLC system. The peptides were subjected to NSI source followed by tandem MS/MS in Q Exactive^TM^ Plus (Thermo) coupled online to the UPLC. Intact peptides were detected in the Orbitrap at a resolution of 70,000 (m/z 200). Peptides were selected for MS/MS using NCE setting as 33 ion fragments were detected in the Orbitrap at a resolution of 17,500 (m/z 200); A data-dependent procedure that alternated between one MS scan followed by 20 MS/MS scans was applied for the top 20 precursor ions above a threshold ion count of 1.5E4 in the MS survey scan with 15.0 s dynamic exclusion. The electrospray voltage applied was 2.0 kV. Automatic gain control was used to prevent overfilling of the ion trap; 5E4 ions were accumulated for generation of MS/MS spectra. For MS scans, the m/z scan range was 350 to 1800. Fixed first mass was set as 100 m/z.

### Database search

The MS/MS data was analyzed using MaxQuant with integrated Andromeda search engine (v.1.4.2). Tandem mass spectra were searched against UniProt_*P. sojae* 6497 (19027 sequences) database concatenated with reverse decoy database. Trypsin/P was specified as cleavage enzyme allowing up to 4 missing cleavages, 5 modifications per peptide and 5 charges. Mass error was set to 10 ppm for precursor ions and 0.02 Da for fragment ions. Carbamido methylation on Cys was specified as fixed modification and oxidation on Met, acetylation on Lys and acetylation on protein N-terminal were specified as variable modifications. False discovery rate thresholds for protein, peptide and modification site were specified at 1%. Minimum peptide length was set at 7. All the other parameters in MaxQuant were set to default values. The site localization probability was set as >0.75.

### Bioinformatics analyses

Gene Ontology (GO) annotation proteome was derived from the UniProt-GOA database (http://www.ebi.ac.uk/GOA) and the proteins were classified by GO annotation based on three categories: biological process, cellular component and molecular function. The annotation protein pathway was performed according to Kyoto Encyclopedia of Genes and Genomes (KEGG)^46^. Functional description of protein domains was annotated by InterProScan based on protein sequence alignment method, and the InterPro domain database was used^47^. Functional annotation tool DAVID was used to identify GO term, KEGG pathway and protein domain^48^,^49^. A two-tailed Fisher’s exact test was used to test specific annotation terms among members of resultant protein clusters. Correction for multiple hypothesis testing was carried out using standard false discovery rate control methods. Any terms with a corrected p-value < 0.05 were considered significant. NetSurfP software was used to predict the secondary structures of proteins^50^. Motif-x software was employed to analyze the model of sequences constituted with amino acids in specific positions of acetyl-21-mers (10 amino acids upstream and downstream of the site) in all protein sequences^51^. Protein-protein interaction networks for the identified acetylated proteins were analyzed by Cytoscape software using interaction data from the PPI database^52^.

## Additional Information

**How to cite this article**: Li, D. *et al*. Acetylome analysis reveals the involvement of lysine acetylation in diverse biological processes in *Phytophthora sojae*. *Sci. Rep.* **6**, 29897; doi: 10.1038/srep29897 (2016).

## Supplementary Material

Supplementary Information

Supplementary Dataset 1

Supplementary Dataset 2

Supplementary Dataset 3

Supplementary Dataset 4

Supplementary Dataset 5

Supplementary Dataset 6

Supplementary Dataset 7

Supplementary Dataset 8

Supplementary Dataset 9

Supplementary Dataset 10

## Figures and Tables

**Table 1:** Acetylated proteins involved in pathogenicity of *P. sojae.*

Protein names	Protein accession	Molecular function	Position	Modified sequence
HSF1	G4Z526	Transcription factors	120	GVFTK(ac)LNTLGETHR
YKT6	G4YEG5	cellular component	5	AVAK(ac)NLLAVR
			72	DVK(ac)WHYIGHVQSNK
VPS1	G4ZPT1	binding	269	EQNFFK(ac)THPAYR
PDI1	G4YXR9	binding	74	LTPEYAAAAK(ac)NLK
			109	GEATLGPLCDGK(ac)QFTLQR
			112	FFK(ac)GDVDAVK
			132	TSAEIEK(ac)WVVK
			206	KDVTEDAAAVNK(ac)VVLYK
			333	YGFDYK(ac)ADDFEAK
			415	ALAPK(ac)YEELAEK
CBEL	G4YIR5	binding	197	QITDK(ac)DYYGNDIK
Avr1d	G4ZLE6	binding	119	AYK(ac)IHYR
PITP1	G5A0N3	binding	169	QTLK(ac)ENCDPATYK

HSF1: The heat shock transcription factor; YKT6: SNARE protein; VPS1: Vacuolar protein sorting gene; PDI1: Protein disulfide-isomerase; CBEL: Cellulose-binding elicitor lectin; Avr1d: Avirulence gene; PITP1: Phosphatidylinositol transfer protein.
