# Supplementary material for: Acetylome analysis reveals the involvement of lysine acetylation in diverse biological processes in Phytophthora sojae
Source: Sci Rep. 2016 Jul 14;6:29897. doi: 10.1038/srep29897 (PMC4944153; doi:10.1038/srep29897)

**Acetylome analysis reveals the involvement of lysine acetylation in diverse biological processes in *Phytophthora sojae***

Delong Li^1,†^, Binna Lv ^1,†^, Lingling Tan^2^, Qianqian Yang^1^, Wenxing Liang^1,*^

^1^The Key Laboratory of Integrated Crop Pest Management of Shandong Province, College of Agronomy and Plant Protection, Qingdao Agricultural University, Qingdao 266109, China. ^2^College of Life Sciences, Qingdao Agricultural University, Qingdao 266109, China. ^†^These authors contributed equally to this work. ^*^Correspondence and requests for materials should be addressed to W.L. (wliang1@qau.edu.cn)

**Figure S1.** Overview of systematic workflow and reliability for the acetylome analysis of *P. sojae*. (a) Overview of experimental procedures used in this study. (b) Mass error distribution of all identified peptides. (c) Peptide length distribution.


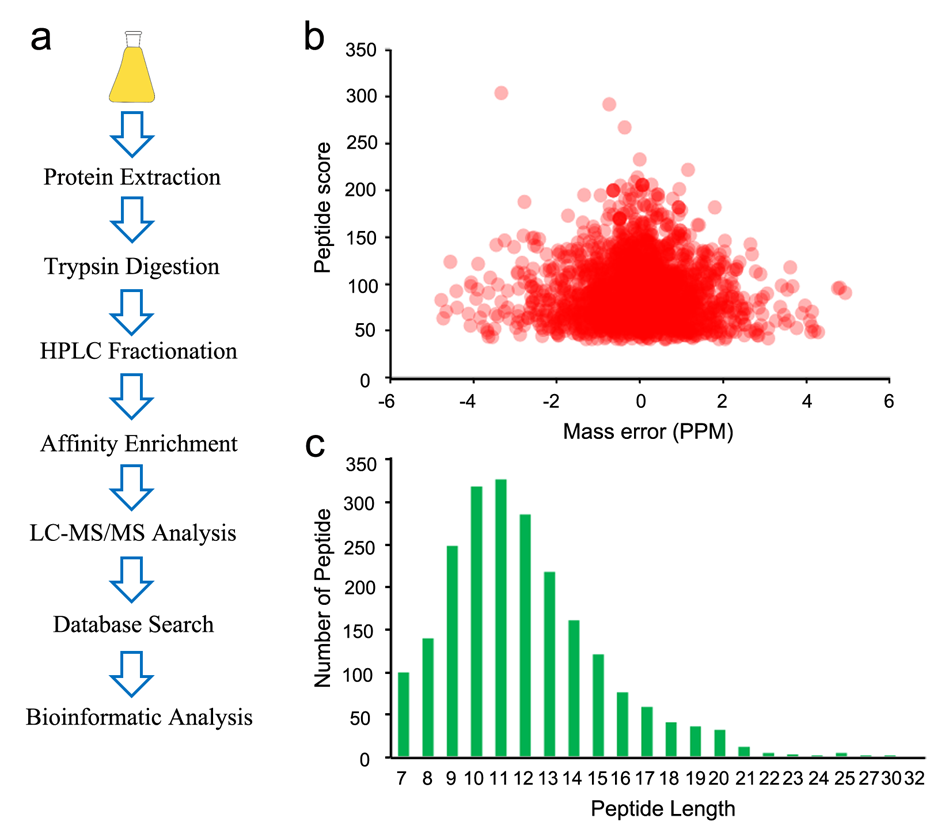


**Figure S2.** Interaction network of acetylated proteins associated with ribosome, proteasome, oxidative phosphorylation and aminoacyl-tRNA biosyntheisis.


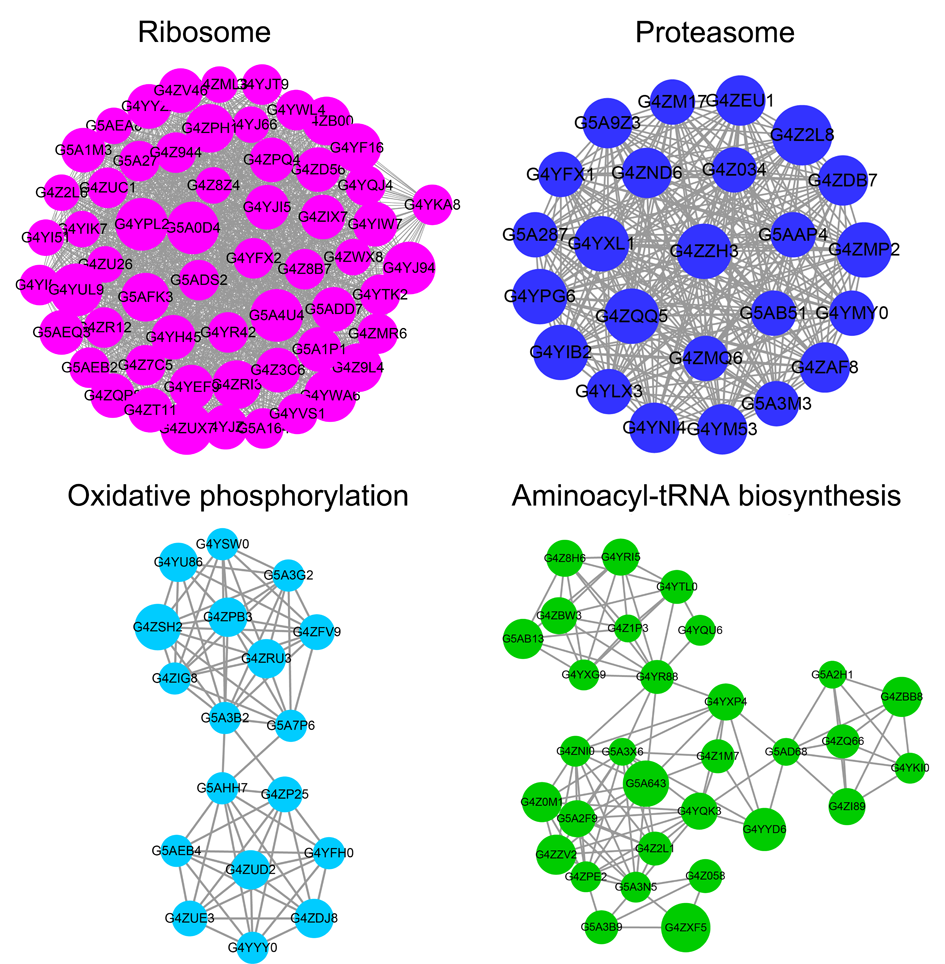

Supplement: Supplementary Information [file srep29897-s1.doc]
